# Effects of Antifungal Carriers Based on Chitosan-Coated Iron Oxide Nanoparticles on Microcosm Biofilms

**DOI:** 10.3390/antibiotics10050588

**Published:** 2021-05-17

**Authors:** Anne Caroline Morais Caldeirão, Heitor Ceolin Araujo, Camila Miranda Tomasella, Caio Sampaio, Marcelo José dos Santos Oliveira, Gordon Ramage, Juliano Pelim Pessan, Douglas Roberto Monteiro

**Affiliations:** 1 Graduate Program in Dentistry (GPD-Master’s Degree), University of Western São Paulo (UNOESTE), Presidente Prudente 19050-920, Brazil; annemcaldeirao@gmail.com; 2 Department of Preventive and Restorative Dentistry, School of Dentistry, Araçatuba, São Paulo State University (Unesp), Araçatuba 16015-050, Brazil; heitor.ceolin@unesp.br (H.C.A.); caio.sampaio@unesp.br (C.S.); juliano.pessan@unesp.br (J.P.P.); 3 School of Dentistry, University of Western São Paulo (UNOESTE), Presidente Prudente 19050-920, Brazil; Camila_Tomasella@hotmail.com; 4 Department of Physics, School of Technology and Applied Sciences (FCT), São Paulo State University (Unesp), Presidente Prudente 19060-900, Brazil; mar.santos.2310@gmail.com; 5 Oral Sciences Research Group, Glasgow Dental School, School of Medicine, Dentistry and Nursing, College of Medical, Veterinary and Life Sciences, University of Glasgow, Glasgow G2 3JZ, UK; Gordon.Ramage@glasgow.ac.uk

**Keywords:** antifungals, biofilms, *Candida*, nanocarriers, iron oxide nanoparticles

## Abstract

Resistance of *Candida* species to conventional therapies has motivated the development of antifungal nanocarriers based on iron oxide nanoparticles (IONPs) coated with chitosan (CS). This study evaluates the effects of IONPs-CS as carriers of miconazole (MCZ) or fluconazole (FLZ) on microcosm biofilms. Pooled saliva from two healthy volunteers supplemented with *C. albicans* and *C. glabrata* was the inoculum for biofilm formation. Biofilms were formed for 96 h on coverslips using the Amsterdam Active Attachment model, followed by 24 h treatment with nanocarriers containing different concentrations of each antifungal (78 and 156 µg/mL). MCZ or FLZ (156 µg/mL), and untreated biofilms were considered as controls. Anti-biofilm effects were evaluated by enumeration of colony-forming units (CFUs), composition of the extracellular matrix, lactic acid production, and structure and live/dead biofilm cells (confocal laser scanning microscopy-CLSM). Data were analyzed by one-way ANOVA and Fisher LSD’s test (α = 0.05). IONPs-CS carrying MCZ or FLZ were the most effective treatments in reducing CFUs compared to either an antifungal agent alone for *C. albicans* and MCZ for *C. glabrata*. Significant reductions in mutans streptococci and *Lactobacillus* spp. were shown, though mainly for the MCZ nanocarrier. Antifungals and their nanocarriers also showed significantly higher proportions of dead cells compared to untreated biofilm by CLSM (*p* < 0.001), and promoted significant reductions in lactic acid, while simultaneously showing increases in some components of the extracellular matrix. These findings reinforce the use of nanocarriers as effective alternatives to fight oral fungal infections.

## 1. Introduction

Polymicrobial biofilms are communities comprising multiple species of microorganisms, including bacteria and fungi, attached to a surface and organized within an extracellular polymeric matrix [1]. In the human body, the presence of structured microbial consortia in biofilms is often observed, modulating the states of health and disease [1]. The oral cavity is considered one of the most favorable environments for polymicrobial biofilm formation, due to its complex features and presence of various retentive niches [1,2], including mucous surfaces, lingual dorsum, tooth hard surfaces, and sub- or supra-gingival compartments [2]. It is thought that the oral microbiome has around 700 different species colonizing this environment [3].

*Candida* species are important contributors for the oral microbiome and may establish a commensal relationship with other microbial species, mainly in healthy individuals [4]. In general, *Candida* yeasts have a high capacity to form biofilms [5] and to induce infections when there are local or systemic disorders, particularly in the immunocompromised [4,5]. Accordingly, a homeostatic imbalance occurs, followed by yeast cell proliferation, establishing a pathological condition [4,5]. Fungal infections affect around one billion people [6] and account for an annual mortality rate of approximately 1.7 million individuals worldwide [6,7]. In such infectious processes, *Candida albicans* stands out as one of the main etiological agents [8,9,10], which is present in about 95% of candidiasis clinical cases [11]. Nonetheless, *Candida glabrata* has been recognized as an important candidiasis-related pathogen in recent years, mainly due to its resistance to antifungal treatments [8] and high prevalence in systemic infections [12].

Among the drugs prescribed to manage candidiasis, miconazole (MCZ) and fluconazole (FLZ) have been frequently used as topical and systemic antifungals, respectively [13,14,15]. Despite the favorable pharmacological properties of these antifungal agents, limitations related to their use have been reported in recent years, encompassing the reduction of antifungal efficacy ought to microbial resistance [16,17,18,19], which hinders the action of drugs and makes them less bioavailable [17,19]. Consequently, administration of higher doses and/or increased frequency are required [19], which may intensify side effects such as a local burning sensation, nausea, vomiting, gastrointestinal disturbances, and hepatotoxicity [11,19]. Another clinical challenge found in the treatment of *Candida* infections refers to the lower availability in the market of antifungals [4,5] compared to antibacterials [5].

Strategies for circumventing the limitations reported above include the study of new alternatives to control fungal infections. In this sense, advances in nanotechnology-based therapies have enabled combining drugs with nanoparticles for improving therapeutic performance of the compound carried, as well as for reducing its side effects [20,21]. Among the numerous nanometric materials available, iron oxide nanoparticles (IONPs) have shown wide application in the biomedical field, including drug delivery, due to factors associated to synthesis process, biocompatibility, chemical stability and surface modification capacity [22,23,24]. As for the latter, chitosan (CS) is a biopolymer with antimicrobial activity successfully used to coat IONPs [21,25,26]. This polymer may establish electrostatic interactions or hydrogen bonds with IONPs [27], favoring the stabilization of nanoparticles under physiological conditions, in addition to allowing the anchoring of drugs [28].

Recently, nanocarriers of MCZ or FLZ assembled from CS-coated IONPs showed similar or superior effects on planktonic cells and biofilms of *C. albicans* and *C. glabrata* compared to those found for each antifungal alone [21,22]. Moreover, we have shown promising effects within orally relevant interkingdom biofilm models, though these were limited to only *C. albicans* and a small panel of preselected oral pathogens [29]. However, these data suggested that targeting the yeast had the potential to destabilize the bacterial components of interkingdom consortia. Therefore, despite these favorable results, the effects of these nanocarriers on *Candida* species in complex polymicrobial interkingdom biofilms remain unknown. We hypothesized that antifungal containing CS-coated IONPs would exert a direct and indirect antimicrobial effect on complex biofilms. Therefore, the aim of the present study is to evaluate the effect of nanocarriers of MCZ or FLZ on undefined microcosm biofilms formed from human saliva supplemented with *C. albicans* and *C. glabrata*.

## 2. Results

### 2.1. Quantification of Cultivable Cells

**Figure 1:**
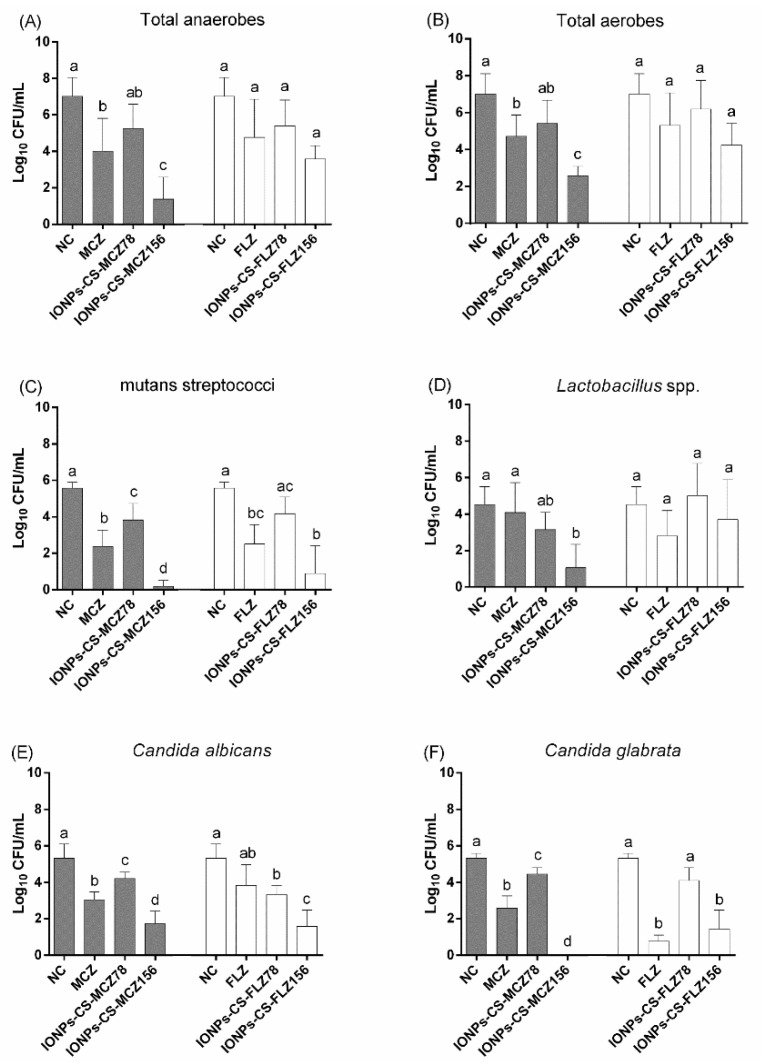
Quantification of colony-forming units (Log_10_ CFU/mL) of total anaerobes (**A**), total aerobes (**B**), mutans streptococci (**C**), *Lactobacillus* spp. (**D**), *Candida albicans* (**E**), and *Candida glabrata* (**F**) from microcosm biofilms formed for 96 h and treated with different compounds. Biofilms were treated during 24 h with miconazole at 156 µg/mL (MCZ), chitosan (CS)-coated iron oxide nanoparticles (IONPs) carrying MCZ at 78 (IONPs-CS-MCZ78) and 156 µg/mL (IONPs-CS-MCZ156), fluconazole at 156 µg/mL (FLZ) and FLZ-containing nanocarrier at 78 (IONPs-CS-FLZ78) and 156 µg/mL (IONPs-CS-FLZ156). Negative control (NC) represents the biofilm formed for 120 h with pure culture medium. Error bars depict standard deviations of the means. Different lowercase letters represent significant differences among the groups (one-way ANOVA and Fisher LSD’s test; *p* < 0.05). Comparisons were performed separately for each antifungal, its respective nanocarrier and the NC.

For total anaerobes and aerobes, MCZ and CS-coated IONPs carrying MCZ at 156 µg/mL (IONPs-CS-MCZ156) were the only treatments that significantly reduced the number of colony-forming units (CFUs) compared to the negative control (NC) (Figure 1A,B). IONPs-CS-MCZ156 was the most effective treatment, which was significantly better than free MCZ, leading to reductions of 5.65- (*p* = 0.046) and 4.43-log_10_ (*p* = 0.037) for total anaerobes and aerobes compared to the NC, respectively.

All compounds significantly decreased the number of CFUs of mutans streptococci compared to the NC (Figure 1C), except CS-coated IONPs carrying FLZ at 78 µg/mL (IONPs-CS-FLZ78). Comparing the effects among each antifungal with their respective nanocarriers, IONPs-CS-MCZ156 was more effective (reduction of 5.38-log_10_; *p* < 0.001) than MCZ (reduction of 3.19-log_10_; *p* < 0.001) in reducing CFUs compared to the NC (Figure 1C). On the other hand, FLZ did not statistically differ from IONPs-CS-FLZ78 and CS-coated IONPs carrying FLZ at 156 µg/mL (IONPs-CS-FLZ156). Regarding the quantification of *Lactobacillus* spp., only IONPs-CS-MCZ156 promoted a significant decrease in CFU number compared to the NC (3.47-log_10_, *p* = 0.009; Figure 1D).

MCZ, CS-coated IONPs carrying MCZ at 78 µg/mL (IONPs-CS-MCZ78) and IONPs-CS-MCZ156 significantly reduced CFU numbers of *C. albicans* and *C. glabrata* compared to the NC (Figure 1E,F). A dose-dependent effect was noted for the nanocarrier of MCZ, with higher reductions promoted by IONPs-CS-MCZ156 compared to IONPs-CS-MCZ78. In addition, IONPs-CS-MCZ156 was the most effective treatment, achieving reductions of 3.6- (*p* < 0.001) and 5.33-log_10_ (*p* < 0.001) compared to the NCs, respectively for *C. albicans* and *C. glabrata* (Figure 1E,F). As for FLZ and its nanocarriers, IONPs-CS-FLZ156 was more effective in reducing the number of *C. albicans* cells than FLZ alone, while for *C. glabrata* these compounds behaved similarly (Figure 1E,F).

### 2.2. Quantification of Extracellular Matrix Components

IONPs-CS-MCZ156 promoted a 5.74-fold increase (*p* = 0.014) in protein content compared to the NC (Table 1). On the other hand, no significant differences among the NC, FLZ, and IONPs-CS-FLZ156 were observed regarding this parameter. As for carbohydrate content, MCZ and IONPs-CS-MCZ156 did not differ from one another, but promoted increases of 18.86- (*p* = 0.041) and 29.44-fold (*p* = 0.006) compared to the NC, respectively. Treatments with FLZ and IONPs-CS-FLZ156 did not differ from one another, but promoted increases of 14.17- (*p* = 0.017) and 14.81-fold (*p* = 0.015) in carbohydrate content compared to the NC, respectively. For DNA content, treatments with MCZ, IONPs-CS-MCZ156 and FLZ resulted in increases of 4.81- (*p* < 0.05), 3.21- (*p* < 0.05) and 4.17-fold (*p* = 0.014) in comparison to the NC, respectively (Table 1).

### 2.3. Quantification of Lactic Acid

**Figure 2:**
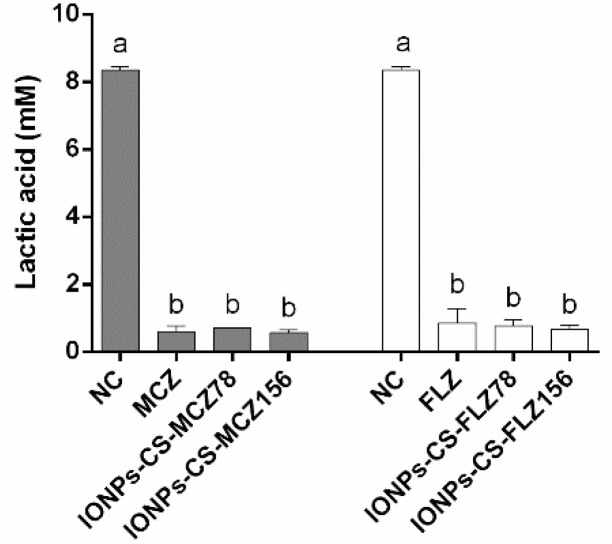
Mean values (standard deviation) of lactic acid concentration from microcosm biofilms formed for 96 h and treated with different compounds. Biofilms were treated during 24 h with miconazole at 156 µg/mL (MCZ), chitosan (CS)-coated iron oxide nanoparticles (IONPs) carrying MCZ at 78 (IONPs-CS-MCZ78) and 156 µg/mL (IONPs-CS- MCZ156), fluconazole at 156 µg/mL (FLZ), and FLZ-containing nanocarrier at 78 (IONPs-CS-FLZ78) and 156 µg/mL (IONPs-CS-FLZ156). The negative control (NC) represents the biofilm formed for 120 h with pure culture medium. Error bars depict standard deviations of the means. Different lowercase letters represent significant differences among the groups (one-way ANOVA and Fisher LSD’s test; *p* < 0.05). Comparisons were performed separately for each antifungal, its respective nanocarrier, and the NC.

MCZ, IONPs-CS-MCZ78, and IONPs-CS-MCZ156 did not significantly differ from one another, but led to significant reductions (ranging from 91.5 to 93.2%; *p* < 0.001) in acid production compared to the NC (Figure 2). The same trend was found for FLZ and its nanocarriers, with significant reductions (*p* < 0.001) in lactic acid production of 89.7%, 90.8% and 91.9% compared to the NC, respectively, for FLZ, IONPs-CS-FLZ78, and IONPs-CS-FLZ156 (Figure 2).

### 2.4. Structural Analysis of Biofilms

**Figure 3:**
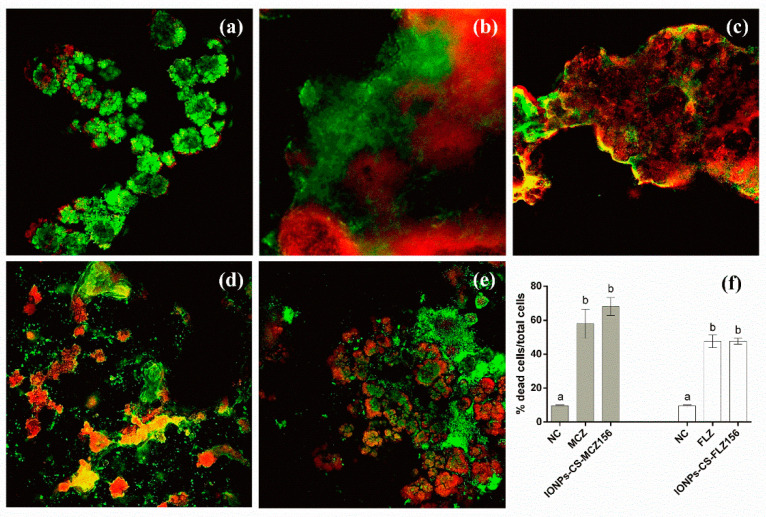
Confocal laser scanning microscopy images of 96-h microcosm biofilms treated during 24 h with miconazole (MCZ) at 156 µg/mL (**b**), chitosan (CS)-coated iron oxide nanoparticles (IONPs) carrying MCZ at 156 µg/mL (**c**), fluconazole (FLZ) at 156 µg/mL (**d**), and FLZ-containing nanocarrier at 156 µg/mL (**e**). The negative control (**a**) represents the biofilm formed for 120 h with pure culture medium. Red and green fluorescence indicate dead and living cells, respectively. Magnification: 20×. The image (**f**) represents the percentage of dead cells in relation to the total cells, and different lowercase letters represent significant differences among the groups (one-way ANOVA and Fisher LSD’s test; *p* < 0.05). Comparisons were performed separately for each antifungal, its respective nanocarrier and negative control.

Confocal laser scanning microscopy (CLSM) images showed biofilms composed by clusters of microbial cells partially covering the surface of the coverslips, regardless of the group evaluated (Figure 3a–e). Treatments with MCZ, IONPs-CS-MCZ156, FLZ, and IONPs-CS-FLZ156 resulted in biofilms with significantly higher proportions of dead cells compared to the NC group (*p* < 0.001; Figure 3f). In turn, IONPs-CS-MCZ156 and IONPs-CS-FLZ156 led to similar reductions in cell viability compared to MCZ and FLZ, respectively (Figure 3f).

## 3. Discussion

Favorable antifungal effects of MCZ and FLZ nanocarriers have been previously reported in studies performed with mono- or dual-species biofilms of *C. albicans* and *C. glabrata* [21,22]. Moreover, we were able to show a positive effect of this chemistry within controlled interkingdom biofilms [29]. Nonetheless, to better mimic the context of the oral microbiome of patients with oral candidiasis, in which *Candida* species are increasing in number and coexisting with other microbial species, the study of microcosm biofilms using saliva from oral candidiasis patients could bring important insights on the benefits of the aforementioned nanocarriers. This, however, was not feasible in the present study, due to the COVID-19 pandemic, which imposed restrictions on dental services worldwide, thus impairing saliva collection from suitable donors. Therefore, the effects of both nanocarriers were evaluated on *C. albicans* and *C. glabrata* in complex ‘real world’ microcosm biofilms. Interestingly, the results of the present study showed that the nanocarriers maintained their effectiveness on *Candida* species, even when they are present and integrated within polymicrobial biofilms with complex architecture.

In general, IONPs-CS-MCZ156 and IONPs-CS-FLZ156 were the most effective treatments in reducing the number of *Candida*, overcoming the effect promoted by each antifungal tested in its free form for *C. albicans* (both nanocarriers) and *C. glabrata* (MCZ nanocarrier) (Figure 1E,F). These findings may be associated with a cooperative action among the three compounds that generate the nanocarriers, so that IONPs functioned as carriers, favoring the penetration of antifungal drugs into the deeper layers of microcosm biofilms. This assumption was previously confirmed, considering that Fe atoms were visible by energy dispersive spectroscopy for elemental mapping in the deeper layers of biofilms treated with nanocarriers based on IONPs-CS [22,26]. In addition, IONPs are able to depolarize the microbial membrane to induce the production of reactive oxygen species and to generate oxidative stress that disturbs cellular homeostasis [24,29], thus contributing to the observed anti-biofilm effects. In turn, the CS coating stabilizes IONPs and facilitates their penetration through the different layers of biofilms, since the positive charge of CS has an electrostatic interaction with the negative charge of the microbial membranes [24,29]. Due to its mucoadhesive property, CS may also contribute to the retention of the nanocarrier in the target cells, in addition to improving the pharmacokinetics and biodistribution of the carried drugs, favoring cell death [24,29]. On the other hand, MCZ and FLZ act on lanosterol 14-α-sterol demethylase, which participates in ergosterol formation (component of the fungal cell wall) [11,30]. MCZ was also shown to promote oxidative stress, due to increases in the production of reactive oxygen species [31].

For *C. glabrata* CFUs, however, IONPs-CS-FLZ156 was not able to overcome the reducing effect generated after treatment with free FLZ (Figure 1F), demonstrating that the effect of this nanocarrier is primarily dependent on the presence of FLZ. *Candida glabrata* has a low intrinsic sensitivity to FLZ [11,32], due to its capacity for specific mutations in the CDR1, CDR2, and MDR1 genes, which are characteristic of azole resistance and are encoded for the action of efflux pumps on the cytoplasmic membrane [30]. Another factor to be highlighted refers to the routes of administration of the different antifungals. FLZ seems to be more relevant for systemic candidiasis (moderate to severe), demanding the use of higher doses to achieve greater effectiveness against *Candida* compared to MCZ, which requires lower doses, due to its topical use [11,33].

It was previously demonstrated that FLZ and IONPs-CS-FLZ did not differ from each other, but promoted significant reductions in the number of CFUs of single biofilms of *Candida* species at 1250 µg/mL [22], which corresponds to ~eight-fold increase over the concentration tested in the present study (156 µg/mL). These findings are extremely relevant from a clinical point of view, as they indicate a superior effect of IONPs-CS-FLZ on *Candida* in polymicrobial consortia, as normally occurs in the oral cavity under pathological conditions. In addition, these results may be indicative of a reduction in the cytotoxic potential of FLZ, since it would make possible the use of lower and more effective doses to combat fungal infections. In contrast, although IONPs-CS-MCZ78 was more effective than free MCZ on *C. albicans* and *C. glabrata* forming mono- or dual-species biofilms [21], this trend was only observed in the present study when the nanocarrier had twice the concentration of MCZ (156 µg/mL). In fact, IONPs-CS-MCZ156 reduced the number of *C. albicans* CFUs by 3.6-log_10_ and completely eradicated *C. glabrata* cells from the microcosm biofilm (Figure 1). Taken together, these findings indicate that the antibiofilm effect is dependent on the concentration of the drug carried and that *Candida* species may be more tolerant to IONPs-CS-MCZ when present in polymicrobial biofilms. This suggestion is corroborated by a recent study showing that the reduction in *Candida* promoted by IONPs-CS-MCZ was accompanied by greater reductions in bacterial cells in three models of pathogenic polymicrobial oral biofilms (gingivitis, denture and caries) [29]. These reductions reflected an increase in the percentage of *C. albicans* in the final composition of the three biofilm models, suggesting that the fungal cells were protected from the action of IONPs-CS-MCZ by bacterial cells [29]. Indeed, Kean and colleagues reported that in dual-species biofilms of *C. albicans* and *Staphylococcus aureus* treated with MCZ, that sensitivity is significantly reduced, supporting the notion of synergistic tolerance in interkingdom biofilms [34].

All compounds evaluated in the present study were also able to significantly reduce the number of CFUs of mutans streptococci compared to the NC, except IONPs-CS-FLZ78 (Figure 1C). These findings may be explained by the interactions established among microorganisms within biofilms. In this context, a symbiotic mechanism between *Streptococcus mutans* and *C. albicans* has been reported in several studies [1,35,36,37], showing that glycosyltransferases (Gtfs) produced and secreted by mutans streptococci promote the breakdown of glucose in monosaccharides, which are metabolized by *Candida* species. This facilitates *Candida* growth and contributes to the production of acids, creating a low-pH environment that helps in the maintenance and survival of *S. mutans* [35,36,37]. In addition, Gtfs may bind to *Candida* cell surfaces and convert sucrose into glucans [35]. These extracellular polymers, in conjunction with the larger surface area of fungal cells (yeasts and hyphae), create propitious conditions for *S. mutans* adherence [37,38]. Specific cell wall receptors (Als3p adhesin) also favor the adherence of other microorganisms to the hyphae of *C. albicans*, including *C. glabrata* [4], *Lactobacillus* spp. and other aerobic and anaerobic bacteria. Consequently, the reductions found for *Candida* species directly influenced the survival conditions of mutans streptococci, impairing their adherence and permanence in the biofilm.

For *Lactobacillus* spp. (Figure 1D), and total anaerobes and aerobes (Figure 1A,B), IONPs-CS-MCZ156 was the most effective treatment in reducing CFUs. These results remain consistent with those previously discussed and reinforce the role of positive interactions between *Candida* and other microorganisms in the colonization, survival, and susceptibility of microcosm biofilms to the compounds tested [39]. Furthermore, although MCZ is a typically antifungal drug, the findings of the present study highlight its antibacterial potential. Probably, free MCZ or conjugated to the core-shell system (IONPs-CS) inhibited bacterial flavohemoglobins, which are responsible for nitric oxide metabolism, resulting in microbial cell death [40,41]. CLSM analysis corroborate these findings, considering that MCZ and IONPs-CS-MCZ156 behaved similarly and presented significantly higher percentages of dead cells than the NC (Figure 3).

On the other hand, FLZ, IONPs-CS-FLZ78, and IONPs-CS-FLZ156 were not able to significantly reduce the number of total anaerobes (Figure 1A), total aerobes (Figure 1B), and *Lactobacillus* spp. (Figure 1D) compared to the NC. A previous study also demonstrated that FLZ was unable to lead to bacterial death [42], corroborating the findings of the present study. Glucans produced by *S. mutans* Gtfs may have sequestered FLZ, limiting its penetration into the biofilm and reducing its effectiveness on bacteria [36,42]. Although the CFU reductions found for *Candida* and *Streptococcus* species after treatment with FLZ and its nanocarriers are in line with CLSM results (Figure 3), such reductions were not reflected in changes in the total microbial load (Figure 1). These discrepancies may be justified by the limitation of the CLSM analysis, which did not represent the entire sample, unlike the CFU analysis. Furthermore, treatments with FLZ and IONPs-CS-FLZ might have favored the development of species that compete for nutrients and binding sites with *Candida* and *Streptococcus*, keeping the total microbial load stable.

In the study reported here, high percentages of lactic acid reduction were found after biofilm treatment with all compounds (Figure 2). The breakdown of glucose precedes the production of lactic acid by bacteria, mainly *Lactobacillus* spp., besides *Streptococcus*, *Enterococcus* and other microbial genera, and plays important roles in the survival and maintenance of these species [43,44]. *Streptococcus mutans*, *S. oralis*, *S. mitis* and *S. gordonii* are all primary colonizers that offer adhesion sites for fungal colonization, in addition to being considered sources of carbon and lactic acid for *Candida* [4]. In turn, *Candida* species in human saliva also contribute to biofilm pH reduction by producing various acids (pyruvate, lactate, and acetate) [45], which favor the activation of acid proteolytic enzymes that damage host tissues. Therefore, the microbial reductions found in the present study directly reflected in decreases in the production of lactic acid by microcosm biofilms. In clinical terms, these results are favorable and highlight that both nanocarriers are capable of affecting an important microbial virulence factor associated with oral candidiasis (acid production).

Regarding the extracellular matrix, there was an overall trend of increases in the values of proteins, carbohydrates, and DNA after treatment with the different compounds, with significant differences between the NC and IONPs-CS-MCZ156 (for all components) and between the NC and IONPs-CS-FLZ156 (for carbohydrates) (Table 1). These increases seem to be related to CFU results, considering that intracellular constituents of dead cells may have been incorporated into the extracellular matrix. Moreover, a higher production of matrix by the remaining biofilm cells may explain these findings, representing an attempt of cellular self-protection against aggression caused by treatments. A previous study demonstrated that FLZ and IONPs-CS-FLZ significantly increased the components of the extracellular matrix of single biofilms of *C. albicans* and *C. glabrata* [22]. In contrast, MCZ alone or conjugated with the nanocarrier acted at the cellular level, without affecting the matrix of mono- or dual-species *Candida* biofilms [21]. These previous results compared to those obtained here emphasize that the nanocarriers’ effects on the extracellular matrix are directly influenced by the type of biofilm analyzed.

It is noteworthy that the use of non-biodegradable nanocarriers may raise concerns on long-term toxicity and bioaccumulation in the organism. Nonetheless, coating of the IONPs with a natural polymer was a procedure used to increase biocompatibility and minimize toxicity [24]. In fact, administration of conventional drugs using nanotechnology may enhance their solubility and pharmacokinetics and reduce immunotoxicity and side-effects [46]. Furthermore, there is evidence that neutrophils and macrophages have important roles on carbon nanotubes biodegradation, by enzymatic digestion of nanoparticles and peroxynitrite-induced oxidation, respectively [46], what might suggest the ability of immunity cells to metabolize other types of nanoparticles. Concerning the potential to promote dysbiosis, although the use of nanocarriers may lead to higher antimicrobial effects compared with its free counterpart, it must be emphasized that antifungals are usually prescribed in clinical situations aiming to reduce the patient’s fungal burden. Within this scenario, dysbiosis may occur as an unwanted side-effect, which may affect the patient’s oral and systemic microbiome. However, it should be highlighted that such effects are transitory, and may occur for both free and conjugated drugs.

In conclusion, IONPs-CS-MCZ and IONPs-CS-FLZ were effective in reducing *Candida* species in salivary microcosm biofilms, surpassing the effects promoted by antifungals in their free form for some of the variables analyzed. Furthermore, significant reductions in the number of mutans streptococci and *Lactobacillus* spp. were found, mainly for IONPs-CS-MCZ. The nanocarriers also promoted significant reductions in the production of lactic acid and increases in some components of the extracellular matrix of microcosm biofilms. Thus, the study’s hypothesis was partially accepted. Future studies evaluating the effects of nanocarriers on microbial ecology (by next generation sequencing) and proteomic profile of the microcosm biofilm, as well as their cytotoxic effects using models of reconstituted human epithelium may contribute to improving the development of antifungal nanocarriers with high sensitivity and selectivity.

## 4. Materials and Methods

### 4.1. Assembly and Characterization of the Nanocarriers

IONPs-CS-MCZ and IONPs-CS-FLZ nanocarriers were obtained by mixing each antifungal with a known concentration of CS-coated IONPs, as previously detailed [21,22]. For characterization, the physico-chemical tests of X-ray diffraction, Fourier-transform infrared spectroscopy, thermogravimetric analysis, transmission electron microscopy, and dynamic light scattering all showed that these antifungal agents were effectively immobilized in the IONPs-CS compound [21,22]. Furthermore, the crystalline structure of the IONPs was not affected after nanocarrier formation, which displayed diameters ≤ 317 nm [21,22].

### 4.2. Candida Strains and Growth Conditions

Two standard strains tested in the present study were purchased from the American Type Culture Collection (ATCC): *C. albicans* (ATCC 10231) and *C. glabrata* (ATCC 90030). Stock cultures were propagated on Sabouraud dextrose agar (Difco, Le Pont de Claix, France) at 37 °C for 48 h. Colonies of each species derived were individually inserted in 30 mL of Sabourand dextrose broth (Difco) and incubated overnight at 37 °C in an orbital shaker (120 rpm). The yeast cells were then centrifuged (8000 rpm, 5 min), washed with phosphate buffered saline (PBS; 0.1 M, pH 7.0), and adjusted in a Neubauer chamber to 1 × 10^7^ cells/mL in human saliva.

### 4.3. Collection of Human Saliva

This study was approved by the local Ethics Committee (CAAE: 22111419.3.0000.5515). Two healthy volunteers (non-smokers) who did not use either oral antimicrobial mouth rinses (over the last 30 days) or systemic antibiotics (over the last 180 days) were selected [47]. Donors also refrained from brushing their teeth on the night before and day of collection and refrained from drinking alcohol in this period. Saliva collection was performed in the morning, at least two hours after eating and/or drinking [47]. Saliva production was stimulated by chewing flexible film (Parafilm^®^ M, Sigma-Aldrich, St. Louis, MO, USA), and the saliva pool from the two donors/volunteers was stored in polypropylene tubes (on ice) [48,49]. The final saliva sample was diluted (1:1) with 60% sterile glycerol and stored at −80 °C until use [49].

### 4.4. Microcosm Biofilm Formation and Treatment with Nanocarriers

Microcosm biofilms were formed on glass discs (coverslips, 12 mm in diameter; Menzel, Braunschweig, Germany) vertically positioned in the Amsterdam Active Attachment model (AAA), as described in detail by Exterkate et al. [49]. Briefly, the saliva pool was diluted (50-fold) in McBain medium [50], whose composition for 1 L of deionized water consisted of 2.5 g mucin (Sigma-Aldrich), 2 g Bacto peptone (Difco), 2 g Trypticase peptone (BBL), 1 g yeast extract (Sigma-Aldrich), 0.35 g NaCl (Sigma-Aldrich), 0.2 g KCl (Sigma-Aldrich), 0.2 g CaCl_2_ (Sigma-Aldrich), 0.1 g cysteine hydrochloride (Sigma-Aldrich), 0.001 g hemin (Sigma-Aldrich), and 0.0002 g vitamin K1 (Sigma-Aldrich) [50], supplemented with 0.2% sucrose (Sigma-Aldrich) *v*/*v* and 50 mmol PIPES (Sigma-Aldrich), at pH 7.0 [49]. *Candida albicans* and *C. glabrata* were added at a final concentration of 1 × 10^7^ cells/mL to the human saliva. This supplementation was performed to mimic a microcosm of oral fungal infections, as well as to ensure the presence of *Candida* species in the polymicrobial biofilm. The inoculum was pipetted (1.5 mL) into each well of a 24-well plate (Falcon^®^; Corning Incorporated-Life Sciences, New York, NY, USA). The plate was closed with the AAA-model lid (containing the coverslips), which was then anaerobically incubated at 37 °C (Anaerobac; Probac do Brasil Produtos Bacteriológicos Ltd.a., São Paulo, Brazil). After 8 h of incubation, the culture medium was replenished by adding 1.5 mL of pure McBain medium in a fresh 24-well plate. This was closed with the same AAA-model lid containing the coverslips with adhered cells. Microcosm biofilms were formed for 96 h, with daily replenishment of the culture medium.

After biofilm formation, the nanocarriers were diluted in McBain medium to reach final concentrations of MCZ and FLZ of 78 and 156 µg/mL, generating two nanocarriers for each antifungal drug: IONPs-CS-MCZ78, IONPs-CS-MCZ156, IONPs-CS-FLZ78, and IONPs-CS-FLZ156. These concentrations were based on the values of minimum inhibitory concentration (MIC) previously published [21], which are equivalent to 50- and 100-fold of the MIC of IONPs-CS-MCZ for *C. glabrata*. For the treatment, the lid of the AAA-model containing 96-h-old biofilms was transferred to a fresh 24-well plate containing 1.5 mL of each nanocarrier suspension, and this was incubated for 24 h. MCZ and FLZ alone, both at 156 µg/mL, were tested as positive controls, while the biofilm exposed to pure McBain medium was considered as the NC.

### 4.5. Quantification of Cultivable Cells

Coverslips with treated biofilms were washed three times with PBS (by transferring the AAA-model lid to 24-well plates containing fresh solutions) to remove weakly adhered cells, and transferred to 5 mL sterile tubes containing 1 mL of PBS. The tubes were placed in an ultrasonic bath for 2 min (55 W; Ultronique, São Paulo, Brazil) and vortexed (1 min). The resulting microbial suspensions were then serially diluted in PBS and plated in the following culture media: (i) Trypticase soy agar (TSA; Difco) with glucose (2 g/L), 5% fresh sheep blood, hemin (10 mL of 0.05% stock solution per liter of medium) and menadione (200 µL of 0.5% stock solution per liter of medium) to count total aerobic and anaerobic microorganisms [49]; (ii) Mitis salivarius agar (MSA; Difco) supplemented with bacitracin (3.3 mg/L), potassium tellurite (1%) and sucrose (15%) for mutans streptococci counts [51]; (iii) Rogosa agar (RA; Difco) supplemented with acetic acid (0.132%) to quantifiy *Lactobacillus* spp. [52]; (iv) CHROMagar *Candida* (Difco) to count *C. albicans* and *C. glabrata*.

TSA plates for total aerobes and CHROMagar *Candida* were aerobically incubated, while TSA plates for total anaerobes were incubated in anaerobiosis. MSA and RA plates were incubated under microaerophilic conditions (5% CO_2_; Microaerobac, Probac do Brasil Produtos Bacteriológicos Ltd.a., São Paulo, Brazil). The number of CFUs (Log_10_ CFU/mL) was counted after 48–72 h of incubation at 37 °C.

### 4.6. Composition of the Extracellular Matrix of Microcosm Biofilms

Coverslips containing the resulting biofilms after treatment were inserted into polypropylene tubes with 2 mL of PBS and vortexed (1 min) to detach biofilms. Afterwards, the tubes were placed in an ultrasonic bath for 2 min (55 W; Ultronique), vortexed for 1 additional min and centrifuged (3000× *g*, 10 min). The supernatant was then filtered through a syringe filter (0.22 µm) to separate the liquid phase of the matrix from the cell pellet [53]. The tubes containing the cell pellets were dried until a constant dry weight was attained, and the difference between this weight and that from the empty tube was considered to be the final dry weight of the biofilm.

The bicinconinic acid method (Kit BCA, Sigma-Aldrich) was used to determine proteins from extracellular matrix, as previously detailed [53]. Briefly, 200 µL of the BCA kit reagent mixture were added to 25 µL of the liquid phase of the extracellular matrix in a 96-well plate (Kasvi, São José dos Pinhais, Brazil). After 30 min of incubation at 37 °C, the absorbance was read at 562 nm, and the standard curve was constructed from known concentrations of bovine serum albumin. In turn, the quantification of carbohydrates was based on the method proposed by Dubois et al. [54], using different concentrations of glucose as standard. A volume of 500 µL of the liquid phase of the extracellular matrix was added to the mixture of phenol with sulfuric acid in glass tubes, which remained at rest for 15 min [53]. Next, the absorbance of the solution was read at 490 nm. For DNA quantification, the absorbance of the liquid phase of the matrix (2 µL) was read on a Nanodrop spectrophotometer (Eon Microplate Spectrophotometer; Bio Tek, Winooski, VT, USA) at 260–280 nm [53]. All data obtained from the matrix components were represented according to the dry weight of the biofilms (mg/g dry weight).

### 4.7. Lactic Acid Production Assay

The wells of a new 24-well plate were filled with 1.5 mL of buffered peptone water (BPW) with 0.2% glucose [49], and the plate was closed with the AAA-model lid after the biofilm treatment period with the nanocarriers. The plate was incubated for 3 h at 37 °C in anaerobiosis, and the lactate concentration in the BPW solution was enzymatically determined (Lactate Dehydrogenase; Sigma-Aldrich) by reading the absorbance at 340 nm, using sodium L-lactate (Sigma-Aldrich) as a standard, ranging from 0 to 10 mM [55]. The values obtained in absorbance/cm^2^ were converted into mM in a Microsoft Excel software spreadsheet (Version 2010, Microsoft Corp., Redmond, WA, USA) to determine the analytical parameters.

### 4.8. Structural Analysis of Biofilms

After 24 h of treatment of the 96-h biofilms with the different compounds, the structural analysis was performed by CLSM. Coverslips containing biofilms were washed with PBS, stained with SYTO9 green fluorescent dye and propidium iodide using the FilmTracer™ Live/Dead™ Biofilm Viability Kit (Invitrogen, Life Technologies Corporation, Eugene, OR, USA), and observed under a confocal microscope (Nikon C2/C2si, Tokyo, Japan), as previously described [21]. Three images from each group were obtained and processed in the ImageJ software (Rasband, W.S., ImageJ, U.S. National Institutes of Health, Bethesda, MD, USA), and the percentages of dead cells were determined by dividing the intensity of red fluorescence (dead cells) by the intensity of green-red fluorescence (total cells).

### 4.9. Statistical Analysis

All biofilm experiments were performed in triplicate, on three different occasions. For the statistical analysis of extracellular matrix components, the results were transformed into a cubic root. All biofilm data showed a normal distribution (Shapiro-Wilk test) and were analyzed by one-way ANOVA and Fisher LSD’s post hoc test (α = 0.05), using the SigmaPlot software (version 12.0; Systat Software Inc., San Jose, CA, USA).

## Figures and Tables

**Table 1:** Mean values (standard deviation) of protein, carbohydrate, and DNA contents extracted from the extracellular matrix of salivary microcosm biofilms treated with miconazole (MCZ) and fluconazole (FLZ), alone or forming nanocarriers.

Matrix Components (mg/g of Biofilm Dry Weight)	Compounds					
	NC	MCZ	IONPs-CS-MCZ156	NC	FLZ	IONPs-CS-FLZ156
Proteins	20.56 (2.38) ^a^	72.16 (41.62) ^a,b^	118.10 (44.23) ^b^	20.56 (2.38) ^a^	65.14 (37.77) ^a^	57.76 (44.73) ^a^
Carbohydrates	35.33 (11.49) ^a^	666.36 (494.17)^b^	1040.32 (145.18) ^b^	35.33 (11.49) ^a^	500.68 (351.48) ^b^	523.30 (320.66) ^b^
DNA	6.75 (0.94) ^a^	32.50 (11.36) ^b^	21.69 (14.78) ^b^	6.75 (0.94) ^a^	28.21 (13.95) ^b^	13.18 (5.53) ^a,b^

Note: for each component of the extracellular matrix, different lowercase letters represent significant differences among the groups (one-way ANOVA and Fisher LSD’s test; *p* < 0.05). Statistical comparisons were performed separately for each antifungal, its respective nanocarrier, and the negative control (NC). Chitosan (CS)-coated iron oxide nanoparticles (IONPs) carrying MCZ (IONPs-CS-MCZ156) or FLZ (IONPs-CS-FLZ156), both at 156 µg/mL.

## Data Availability

The data presented in this study are available on request from the corresponding author.
